# Advances in Neurological Diseases and Stroke

**DOI:** 10.3390/jcdd10020066

**Published:** 2023-02-04

**Authors:** Narayanaswamy Venketasubramanian

**Affiliations:** Raffles Neuroscience Centre, Raffles Hospital, Singapore 188770, Singapore; ramani_nv@rafflesmedical.com

Neurological diseases are a major cause of death and disability worldwide. Based on the Global Burden of Disease Study (GBD), in 2016, neurological disorders were the leading cause of disability-adjusted life years lost (DALYs) (276 million, 95% UI 247-308) and second-leading cause of death (9.0 million, 95% UI 8.8-9.4), with stroke being the largest contributor to the DALYs (42.2%, 95% UI 38.6-46.1) [1]. This Editorial will highlight some of the key findings of papers on neurological diseases published in this Journal from 2020 to 2022.

Mitochondrial diseases are due to mutations in mitochondrial or nuclear DNA that affect adenosine triphosphate (ATP) production. High energy-consuming organs are most frequently involved, such as the brain, skeletal muscle and heart. Neurological syndromes include chronic progressive external ophthalmoplegia (CPEO), Kearns Sayre syndrome (CPEO with pigmentary retinopathy), MELAS (mitochondrial myopathy MM, encephalopathy, lactic acidosis, and stroke-like episodes), and MERRF (myoclonus epilepsy with ragged red fibers). In a systematic review of cardiac involvement in MM that included 825 participants in 21 studies, 39% had conduction abnormalities on electrocardiogram (ECG) and 29% had cardiac structural abnormalities on echocardiogram, with more severe cardiac phenotypes in MERRF and MELAS [2]. In a paper by Nikhanj et al., 53 patients with mitochondrial diseases and neurological symptoms, median age 50 years, 64.2% female, were enrolled [3]. Over a 4-year period, 11.3% were diagnosed as having heart disease while 5.7% had cardiomyopathy. The authors felt that the low prevalence of cardiomyopathy in their study showed there was a range of phenotypic presentations in MM, and they stressed the presence of comorbidities, such as aging and risk factors, for cardiovascular diseases in their cohort.

Hypertension is the strongest modifiable risk factor for stroke. Primary aldosteronism (PA), due to renin-independent autonomous secretion of aldosterone, is found in 5 to 10% of hypertensive patients [4]. A meta-analysis of 31 studies that included patients with PA (*n* = 3838) and essential hypertension (EH, *n* = 9284) showed that compared to patients with EH, those with PA had a higher risk of stroke, coronary artery disease, heart failure and atrial fibrillation [5]. However, it is unclear what is the best management for PH in reducing the risk of stroke. Qian et al. performed a systematic review and meta-analysis of three studies involving patients with PA undergoing medical treatment (*n* = 3244) or adrenalectomy (*n* = 1611), compared to patients with EH (*n* = 20,568); mean age of PH patients was 49.85 years, 40–54% female [6]. Compared to medical treatment, PA patients who underwent adrenalectomy had a reduced risk of stroke (OR: 0.57, 95% CI 0.35–0.93, *p* = 0.03). The authors concluded that PA is a modifiable risk factor for stroke, especially via adrenalectomy.

A meta-analysis of 32 studies involving 10,892 patients showed that high blood pressure (BP) in acute ischemic stroke (AIS) or primary intracerebral haemorrhage (PICH) is associated with subsequent death, death or dependency, or death or deterioration, depending on whether it is mean, systolic or diastolic BP (DBP) [7]. These studies were not performed in a telestroke network, and generally did not evaluate the effect of risk factors and their interaction with levels of DBP. Pre-stroke factors, such as morbidities, diet, medication and demographics, may also affect the severity of a stroke [8]. Brown et al. performed a retrospective analysis of 452 AIS patients in a telestroke network, mean age 64 years, 51.3% female [9]. Elevated DBP was defined as >80 mmHg, while increased stroke severity was NIHSS > 7. For DBP ≤ 80 mmHg, increased heart rate was associated with higher stroke severity (OR = 1.025, 95% Cl, 1.001–1.050, *p* = 0.042). For DBP > 80 mmHg, increased stroke severity was associated with hypertension (OR = 3.453, 95% Cl 1.137–10.491, *p* = 0.029), history of smoking (OR = 2.55, 95% Cl 1.06–6.132, *p* = 0.037) and heart rate (OR = 1.036, 95% Cl 1.009–1.064, *p* = 0.009). The authors highlighted specific risk factors among AIS patients with elevated DBP that may need to be managed.

Clinical trials have convincingly shown that statins are able to reduce the risk of major vascular events in both women and men [10]. A meta-analysis of 17 studies with 158,353 patients showed that pre-stroke statin use was significantly associated with mild stroke; pooled data from 7 studies provided an OR 1.24 (95% CI, 1.05–1.48; *p* = 0.013) [11]. This benefit has been attributed to more collaterals and better reperfusion. Mori et al. reported the results of their retrospective study within a prospective stroke registry of 1110 propensity-matched AIS patients, mean age 79 years, 43% female [12]. They found that pre-stroke statin use was associated with mild neurological deficit (NIHSS ≤ 3) (*p* = 0.0385). This was despite the very low doses of statin used (<10 mg/dy). An added benefit was a higher proportion being discharged home (52.1% vs. 45.6%, *p* = 0.0306). Overall, the results of this study are consistent with the previous meta-analysis and provide an added reason for statin prescription among patients at risk of vascular events.

Mechanical thrombectomy for patients with AIS and accessible large artery occlusion who can be treated within 6 h of stroke onset increases the odds of a favourable outcome by about 37% [13]. Unfortunately, there may be a failure of recanalisation or early re-occlusion. A meta-analysis of seven randomised controlled trials and observational studies involving 1175 patients given tirofiban, a GPIIb/IIIa receptor antagonist, or control, showed reduced peri-operative re-occlusion, but did not show better recanalisation or 3-month functional outcome, nor increased symptomatic intracranial haemorrhage (ICH) [14]. Cai et al. performed a retrospective study of 285 patients who underwent thrombectomy for anterior circulation large artery occlusion, mean age 71 years, 61.7% female [15]. The clinical decision for giving tirofiban was left with the attending physician. They found that tirofiban was associated with a higher rate of favourable outcomes (mRS 0–2) (OR = 2.033, 95% CI 1.002–4.123, *p* = 0.043), without an increased risk of ICH or death. Subgroup analyses showed that favourable outcomes were among patients with NIHSS > 14 (OR 2.778, 95% CI 1.056–7.356, *p* = 0.038). Tirofiban may, thus, be more beneficial among those with more severe stroke.

Cryptogenic stroke (CS), where the cause is unknown despite investigation, comprises 30% to 40% of ischemic strokes [16]. Some CS may actually be due to atrial fibrillation (AF) that was not detected during routine 24-h Holter monitoring, but which may be picked-up with longer monitoring techniques including implantable loop recorders (ILR) [17]. Unfortunately, ILPs carry the problems of cost, patient acceptability and complications. Kulach et al. performed a cohort study of 78 patients, mean age 60.3 years, 42.3% female, to evaluate the value of 7-day Holter monitoring to detect stroke-causing arrhythmias among CS patients whose 24 h Holter was free of arrhythmia [18]. They found supra-ventricular (SV) runs (≥5 QRS) in 36% and AF in 9%. During the 3 years of follow-up, those with SV runs during the 7-day Holter had a higher incidence of AF than those without (RR 11.6, 95%CI 2.82–47.4, *p* = 0.0007). They thus suggested that 7-day Holter monitoring be performed among those with negative baseline 24-h monitoring, and those with SV runs be considered for extended continuous monitoring.

Aspirin is recommended in clinical practice guidelines for the secondary prevention of vascular events, such as recurrent stroke, myocardial infarction or vascular death, after transient ischaemic attack (TIA) or ischemic stroke (IS) [19,20]. Aspirin resistance (AR) is said to be present if there are recurrent events among aspirin-treated patients—this may be due to medication non-compliance, inadequate dosing, disease mechanism not responsive to aspirin, such as cardioembolism and genetic polymorphism, to name a few. A meta-analysis of 20 studies with 4989 patients reported high on-treatment platelet reactivity to aspirin in 3–65% [21]. Despite the wide range of tests available to detect AR, the ‘best test’ is still unclear [22]. Venketasubramanian et al. reported the results of their cohort study of 113 patients, mean age 65 years, 47% female. AR was found in 44.3% on at least one of four tests [23]. While the identification of AR was inconsistent across different platelet function tests, the strongest correlation was between light transmission aggregometry using arachadonic acid (LTA AA), VerifyNow^®^ and Multiplate^®^ ASPItest. The best predictor of vascular outcome was LTA of ≥70% using 10 µM ADP.

A meta-analysis of almost 380,000 patients from 20 studies found that only 57% were adherent with taking their medications for primary or secondary prevention of cardiovascular disease (CVD) [24]. Good adherence reduces the risk of fatal or non-fatal coronary heart disease, stroke or sudden cardiac death [25]. Liu et al. performed a meta-analysis of 46 articles to assess for a dose response between CV medication adherence, CV events and all-cause mortality [26]. Data were available for 4,051,338 unique patients, average age 60.1 years, 44% female. Over an average follow-up of 4.2 years, they found that a 20% increase in adherence level to cardiovascular, antihypertensive and lipid-lowering medications was associated with significant reductions in the study end-points, ranging from 7 to 9% for CV events, 13 to 17% for stroke and 9 to 12% for all-cause mortality. They felt that priority should, thus, be given to the development of cost-effective measures to increase medication adherence.

Cardiac surgery and vascular procedures may be critical and life-saving procedures. However, they carry the risk of neurological complications, such as stroke, seizures, delirium, cognitive dysfunction and neuropathies [27]. These complications may prolong length of intensive care unit and hospital stay, cause functional impairment and increase the cost of medical care, with negative effects on patients [28]. Teller et al. reported the results of their prospective study describing the various neurological complications after cardiac surgery or percutaneous valve replacement among 297 patients, median age 74 years, 37% female [29]. During the first 3 post-operative days, they found neurological complications in 43.8%, comprising delirium (43.43%), stroke (2.7%), seizures (1.35%) and hallucinations (3.36%). Complications were associated with lower Montreal Cognitive Assessment (MoCA) scores (Exp(B) 2.042; 95% CI, 1.183–3.525, *p* = 0.010), older age (Exp(B) 1.071; 95% CI, 1.036–1.107, *p* < 0.001), red blood cell transfusions until postoperative day 3 (Exp(B) 1.157; 95% CI, 1.030–1.300, *p* = 0.014), history of heart failure (Exp(B) 1.985; 95% CI, 1.130–3.487, *p* = 0.017) and increased CRP levels (Exp(B) 1.004; 95% CI, 1.000–1.008, *p* = 0.037). This study suggests that those with risk factors should be closely monitored for post-operative neurologic complications.

Nephrotic syndrome in children is usually responsive to steroids. For those who become steroid-resistant, options include cyclosporine, tacrolimus and mycophenolate mofetil [30]. Sakr et al. reported on a case of a 32-month-old child with relapsing nephrotic syndrome that was initially responsive to steroids but became non-responsive and was complicated by cerebral venous thrombosis [31]. The child responded to a combination of pulsed intravenous methylprednisolone, enoxaparin and rituximab, which is a chimeric monoclonal antibody that binds to the CD20 antigen on the surface of B-lymphocytes and depletes it.

These publications, ranging from meta analyses to case-control studies to case series to a case report, provide new knowledge on neurological diseases, post-operative complications, factors affecting stroke severity, acute stroke treatment, stroke mechanisms and secondary prevention. These will provide valuable information that may assist clinicians in better management of their patients. They also raise pertinent issues that will sow the seeds for future impactful research into neurological diseases.
